# Early Recognition and Intervention in SIBlingS at High Risk for Neurodevelopment Disorders (ERI-SIBS): a controlled trial of an innovative and ecological intervention for siblings of children with autism spectrum disorder

**DOI:** 10.3389/fped.2024.1467783

**Published:** 2025-01-06

**Authors:** Silvia Annunziata, Giulia Purpura, Elena Piazza, Paolo Meriggi, Gabriele Fassina, Laura Santos, Emilia Ambrosini, Antonella Marchetti, Federico Manzi, Davide Massaro, Andrea Luna Tacci, Elisabetta Bolognesi, Simone Agostini, Francesca La Rosa, Alessandra Paola Giulia Pedrocchi, Paola Molina, Anna Cavallini

**Affiliations:** ^1^ IRCCS Fondazione Don Carlo Gnocchi, Milano, Italy; ^2^ School of Medicine and Surgery, University of Milano Bicocca, Monza, Italy; ^3^ Department of Electronics, Information and Bioengineering, Politecnico di Milano, Milano, Italy; ^4^ UniToM, Università Cattolica del Sacro Cuore, Milano, Italy; ^5^ Department of Psychology, Università Cattolica del Sacro Cuore, Milano, Italy; ^6^ Interuniversity Department of Regional and Urban Studies and Planning (DIST), University of Turin, Turin, Italy

**Keywords:** early intervention, autism spectrum disorder, neurodevelopmental disorders, siblings, children

## Abstract

**Background:**

It has been widely demonstrated that siblings of children with autism spectrum disorder (ASD) have an increased risk of abnormal developmental trajectories. In response to this, early recognition protocols have been developed worldwide, aiming to promote early interventions that can positively impact the neurodevelopment of this population. This paper presents the protocol of a controlled trial: ERI-SIBS (Early Recognition and Intervention in SIBlingS at High Risk for Neurodevelopment Disorders) is an innovative and ecological early recognition and intervention program designed specifically for siblings of children with ASD.

**Methods:**

We aim to recruit siblings at low risk and high risk of neurodevelopmental disorders. Based on clinical evaluation at T0, we will allocate the infants into three groups: Group 1, infants at low risk without any signs of neurodevelopmental disorders; Group 2, infants at high risk without any signs of neurodevelopmental disorders; Group 3: infants at low or high risk with signs suggestive of neurodevelopmental disorders. Children of Group 2 will undergo Active Monitoring (one 90 min session once a month for 6 months), while children of Group 3 will undergo Early Intervention (one 90 min session once a week for 6 months). In both cases, the ERI-SIBS contents are based on a multidimensional and naturalistic approach and always involve caregivers. All recruited children will be evaluated at three different time points (T0 within the 8 months of life of the child, T1 after 6 months and T2 after 12 months) using behavioural, technological, and biological techniques to assess infants’ neurodevelopmental functions, parent-infant interaction, and early ASD markers.

**Discussion:**

The ERI-SIBS study will expand knowledge regarding the impact of early intervention on families of infants at risk of neurodevelopmental disorders for the presence of a child with a diagnosis of ASD. The study will have the potential to significantly contribute to future research and the scientific and clinical debate on the best way to implement early intervention in at-risk populations.

**Clinical Trial Registration:**

Clinicaltrials.gov identifier (NCT06512649).

## 1 Introduction

Autism spectrum disorder (ASD) is a neurodevelopmental disorder that is diagnosed in an average of 1 in every 36 8-year-old children in the U.S., and its prevalence has been growing over the last few decades (1). The mechanisms underlying ASD remain largely unknown. However, a genetic contribution is supported by twin studies (2) and by evidence that siblings of ASD patients are at greater risk for ASD or other neurodevelopmental disorders than those of the general population (3). The heritability of ASD is estimated to be 50%, captured mainly by still unknown common variants (4). However, it has also been shown that siblings of ASD patients are at increased risk of abnormal developmental trajectories, even in the absence of a specific diagnosis (5). Recently, Di Giovanni et al. (6) suggested that several genes that can act and/or interact in different pathways are associated with different ASD phenotypes, confirming that epigenetic mechanisms may also play a key role in ASD. Moreover, recent studies on ASD families (including unaffected siblings) highlighted the importance of an immunogenetic pattern involved in alterations of the embryo-foetal environment, maternal immune activation (MIA) and postnatal mechanisms of neuroinflammation (7, 8).

In this scenario, early diagnosis and identification of at-risk categories are crucial. Bradshaw and colleagues reported that observable social-communication differences for infants with ASD are unfolding by 9 months, suggesting that this time may be considered a critical window for targeted intervention (9). Several observational studies worldwide have focused not only on monitoring at-risk populations and identifying early ASD predictors in newborns and infants but also on the management of medical and psychiatric comorbidities in these children (10–12). Indeed, recognising predictors and prodromes of neurodevelopmental disorders early in life should allow prompt referral to treatment. As a matter of fact, it has already been demonstrated that starting treatment within the first two years of life can positively impact developmental trajectories (13). Recently, Guthrie and colleagues showed that early intervention started at 18 months of age, compared to 27 months, significantly improved receptive and expressive language and social communication skills (14). Although the importance of early diagnosis is widely recognised in the medical literature, scientific evidence about the effects of early intervention on developmental outcomes is still very limited (15–17). This emergent area of research focuses on the application of several types of intervention already in the first year of life on children's populations considered epidemiologically “at risk” since siblings of a child with an ASD diagnosis and in whom there is a presence of suggestive signs for a neurodevelopmental disorder. For example, Green and collaborators (15) assessed the effect of a developmental intervention (Video Interaction to Promote Positive Parenting—iBASIS-VIPP) for infants at high risk of ASD. It was mediated by parents and was designed to promote play and positive interactions between the child and caregiver. Specifically, the aim of this was to improve the quality of parents’ understanding of the infant's communication; therefore, first, the focus was on interpreting the infant's behaviour and recognising their intentions, followed by working on sequences of sensitive responses during everyday activities, emotional synchronisation, and patterns of verbal and nonverbal interaction. The authors showed positive findings across a wide range of behavioural and brain function risk markers and developmental outcomes coherently with a moderate intervention effect to reduce the risk for later autism.

Moreover, these data are consistent with findings that support the possibility of modifying neural circuitry and, consequently, functional processes through environmental enrichment in early life, providing a combination of multisensory/cognitive stimulation, increased motor activity, and enhanced social interactions and eliciting natural explorative behaviours (18). Early interventions inspired by the environmental enrichment paradigm were ecological, non-invasive, and well thought-out to enhance neuroplasticity through experience. Several research groups have investigated the profound and positive effects of intensive and multisensory stimulation during the early stages of life on the human brain and on children at risk of developing neurodevelopmental disorders (19–21). Based on these findings, Whittingham and colleagues (22) recently published the ENACT protocol (ENvironmental enrichment for infants; parenting with Acceptance and Commitment Therapy), which was the first randomised controlled trial to test a very early intervention for infants at risk of ASD (infants with one or more biological siblings or a biological parent diagnosed with ASD). This model is characterised by the application of a combination of parent-mediated very early intervention with parental mental health support within the first six months of life of high-risk infants.

Remarkably, there are no specified and shared protocols in Italy in this field, although an Italian-published case report recently suggested the importance of a pre-emptive approach (23). Based on these issues, we aimed to verify the feasibility of an early, naturalistic, parent-mediated intervention in siblings of ASD children within the Italian rehabilitation context and to analyse the effects of this model on their developmental trajectories. To study this issue, we will recruit infants at low risk since siblings of children with typical development (TD) and infants at high risk because of siblings of children affected by ASD.

Based on clinical evaluation at T0, we will divide the infants into three groups as follows:

• Infants at low risk without any signs of neurodevelopmental disorders (Group 1)
• Infants at high risk without any signs of neurodevelopmental disorders (Group 2)
• Infants at low or high risk with signs suggestive of neurodevelopmental disorders (Group 3).

### 1.1 Hypothesis

#### 1.1.1 Primary outcome

**H1:** The main hypothesis of this study is that early parent-mediated intervention in children at risk for neurodevelopmental disorders can be implemented in routine care, positively impacting early sensory-motor and socio-communicative developmental trajectories and reducing the developmental gap in children with signs of concern. For this first aim, we will use standardised, age-appropriate, and sensitive tools to define the presence of early signs suggestive of neurodevelopmental disorders and to monitor their developmental trajectories over time.

#### 1.1.2 Secondary outcomes

**H2:** Here, it is postulated that early intervention with active parental involvement can reduce parental stress and improve parental understanding of and responsiveness to a child's communication cues. This hypothesis will be tested by standardised tools that consist of scoring parental style of interaction during a free-play sequence and intercepting stress signals by an auto-administered questionnaire for parents.
**H3:** An important part of our work will be analysing data about early social and joint attention behaviours in recruited children and comparing them at different time points. The hypothesis is to identify early differences between groups at baseline and to detect changes before and after the intervention. For this reason, in our study design, we decided to use technologies to collect data on quantitative measures during play-structured and laboratory sessions to understand changes in developmental trajectories.
**H4:** Given the potential role of genetic and immunological mechanisms in ASD, one of the secondary aims of this study is to investigate the impact of an early intervention programme on epigenetic changes and inflammatory and immune responses. This hypothesis is based on the possibility that behavioural symptoms are related to genetic markers and biological mechanisms underlying the aetiology of ASD. It is, therefore, conceivable that certain risk biomarkers for ASD or other neurodevelopmental disorders may be present in these populations. Identifying such biomarkers may improve our understanding of genetic risk factors and biological processes underlying these conditions.

## 2 Methods and analysis

### 2.1 Trial design

The study is a controlled trial following the Consolidated Standards of Reporting Trials (CONSORT) guidelines. After enrolment and baseline assessments (T0), the children will be allocated to one of three groups:

Group 1—Clinical Monitoring Group (CM): Siblings of TD children with no signs of concern
Group 2—Active Monitoring Group (AM): Siblings of ASD children with no signs of concern
Group 3—Early Intervention Group (EI): Siblings classified as “with signs of concern” at the baseline evaluation.

**Figure 1:**
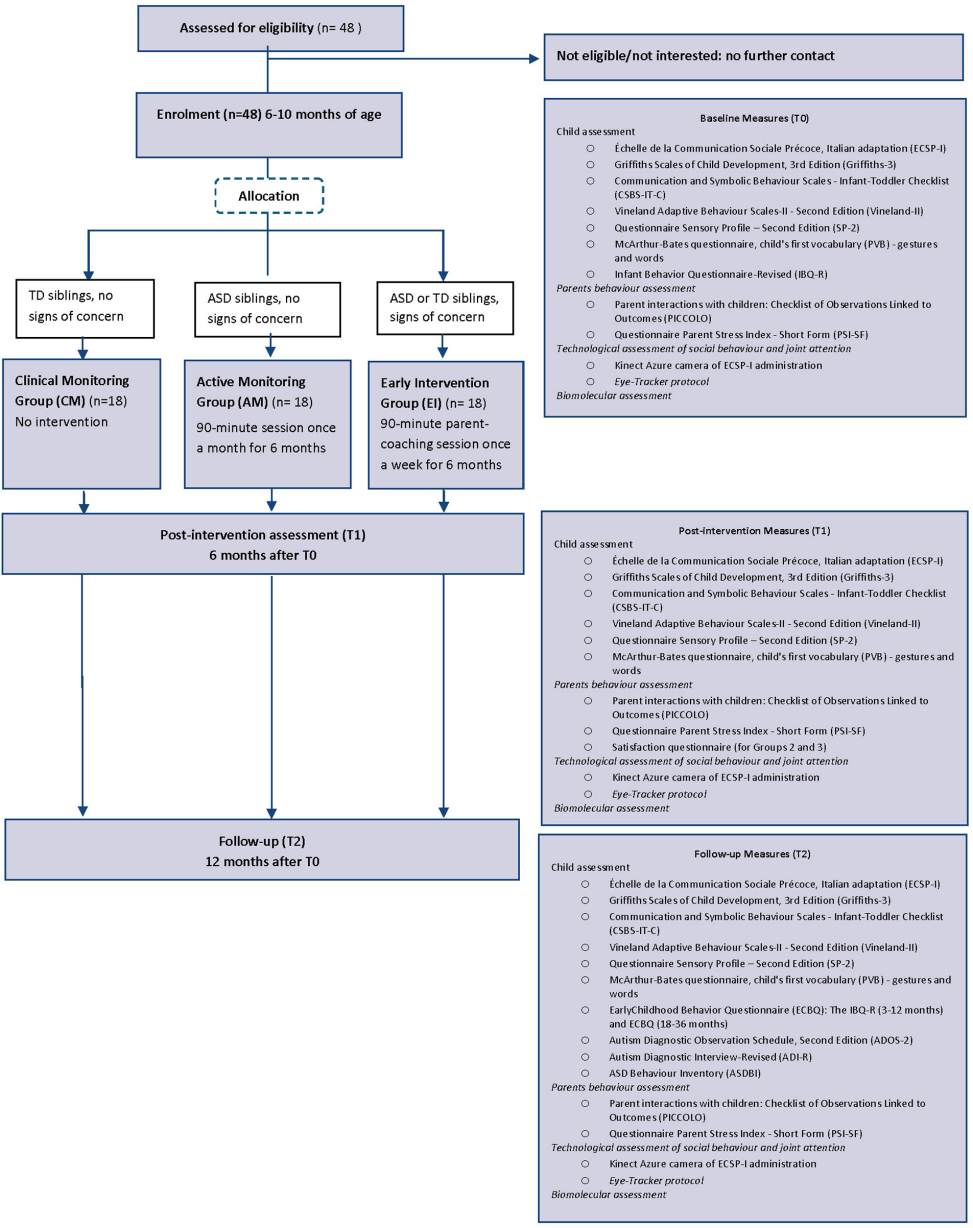
CONSORT flow chart of the ERI-SIBS (early recognition and intervention in SIBslings at high risk for neurodevelopmental disorders) study.

All children will be re-evaluated after 6 months (T1) and 12 months (T2) from T0. Figure 1 depicts the CONSORT flow chart.

### 2.2 Recruitment

Families will be recruited via advertisements distributed through several Fondazione Don Gnocchi (FDG) branches in Lombardia, Italy. The study will be performed in the most important department of Fondazione Don Gnocchi, which is the scientific institute IRCCS Santa Maria Nascente in Milano; this is one of the leading Italian healthcare centres for the diagnosis and rehabilitation of children with neurodevelopmental disabilities. Siblings of TD children will be recruited with the help of daycares in the same area. All families will be recruited during pregnancy or until the infant reaches 8 months of life and will be followed over the following 12 months.

### 2.3 Inclusion criteria

Participants have to meet the following inclusion criteria: (1) the infant must have one or more biological siblings; (2) the child must not be more than ten months of age at the time of the first assessment; (3) the child must undergo a normal neurological examination; (4) parents must agree to the assessment requirements; and (5) the parents must have sufficient Italian language to complete the assessments.

### 2.4 Exclusion criteria

The exclusion criteria are as follows: (1) any infant with known neurological or chromosomal disorders or with suspected genetic syndromes at the time of recruitment; (2) parents who did not speak Italian fluently; (3) children already in behavioural treatment for any developmental disorders.

### 2.5 Outcome measures

#### 2.5.1 Neurodevelopmental assessment

##### 2.5.1.1 Main outcome measures

• Early Social Communication Scale (Échelle de la Communication Sociale Précoce, Italian adaptation, ECSP-I): The ECSP-I is a structured assessment to measure individual differences in nonverbal communication skills in children from 3 to 30 months of age. The administration requires 15–20 min and includes the presentation of approximately 23 standardised situations that provide opportunities for social communication (24). The ECSP-I demonstrated strong reliability and both construct and discriminant validity.
• Griffiths Scales of Child Development, 3rd Edition (Griffiths-3): Griffiths-3 is the gold standard for providing an overall measure of a child's development (0–6 years); it permits the definition of an individual profile of the child's strengths and needs across five areas: foundations of learning, language and communication, eye-hand coordination, personal-social-emotional, and gross motor (25).
• The Communication and Symbolic Behaviour Scales—Infant-Toddler Checklist (CSBS-IT-C) (26) is a developmental screening tool to measure 7 language predictors in children aged 6–24 months. The questions of the checklist may be presented in an interview format with adequate explanations to clarify what is being asked. It takes approximately 5–10 min to complete and permits the detection of signs of concern for ASD in infants and toddlers.
• Satisfaction Survey: The parents of children in Groups 2 and 3 will be asked to anonymously complete a survey (created *ad hoc* for this project) immediately after the intervention (T1) to assess their satisfaction with the services provided. The questionnaire took 5 min to complete.

##### 2.5.1.2 Secondary outcome measures

• Vineland Adaptive Behaviour Scales-II—Second Edition (Vineland-II): This assessment measures everyday life's personal and social adaptation skills. It applies to children from birth through 18 years and 11 months. The standardisation included national samples of children with and without disabilities. The scales yield normative standard scores that indicate the level of adaptive functioning (27).
• Questionnaire Sensory Profile—Second Edition (SP-2): The SP-2 is a norm-referenced collection of five parent- and teacher-report questionnaires that assess sensory processing in children about everyday sensory events. For this study, the age-appropriate version was used, the Toddler Module (from 7 to 35 months); items are rated on a 5-point Likert scale from 5 (almost always) to 1 (almost never) (28, 29).
• McArthur-Bates questionnaire, child's first vocabulary (PVB)—gestures and words: The PVB assesses major features of communicative development, including word comprehension and production, gesture use, and first sentence comprehension. This tool is a checklist completed by the parents that has been validated on an Italian sample, and it allows us to calculate the quotients related to the different subareas investigated (30).
• Infant Behavior Questionnaire-Revised (IBQ-R) and Early Childhood Behavior Questionnaire (ECBQ): The IBQ-R (3–12 months) and ECBQ (18–36 months) are parent-report questionnaires for assessing temperament in infancy and later childhood; items are rated on a 7-point scale (from 1, which is never to 7, which is always) (31, 32).
• Autism Diagnostic Observation Schedule, Second Edition (ADOS-2): The ADOS-2 is a semi-structured, standardised assessment of communication, social interaction, play/imaginative use of materials and restricted/repetitive patterns of interest to assess the presence of ASD symptoms. The administration involves direct observation using hierarchical manualised procedures and progressive prompts. Every behaviour/symptom is assessed using a Likert scale (0–3) and coded on an algorithm based on DSM-5 diagnostic criteria. We will use the Toddler Module for 12–30-month-old children or Module 2 if language in sentences is present only at T2. We will consider the total score, social affect score, and restricted and repetitive behaviours score (33).
• Autism Diagnostic Interview-Revised (ADI-R): The ADI-R is a semi-structured interview conducted by a trained researcher or psychologist with the child's parents or other caregivers, based on DSM-IV criteria. It focuses on the child's developmental history and the actual description given by the parents. Every behaviour/symptom is assessed using a Likert scale (0–3) and coded using an algorithm. We will use algorithms developed explicitly for toddlers that provide a clinical cut-off and a risk rate for ASD at T2 (34, 35).
• ASD Behaviour Inventory (ASDBI): The ASDBI is the Italian version of the Pervasive Developmental Disorder Behavior Inventory—PDDBI (36). It is a questionnaire that provides information to monitor signs of autistic behaviour in several domains: sense/perceptual contact models, ritualism/resistance to change, pragmatic/social problems, semantic/pragmatic problems, difficulties of excitability, specific fears, aggression, behaviour in social relationship, expressive language, learning and memory. For this study, we will use the Italian version of the short form of ASDBI at T2.

#### 2.5.2 Parents’ behaviour assessment

• Parent Interactions with Children: Checklist of Observations Linked to Outcomes (PICCOLO): This is an observational measure in which a parent-child interaction is video recorded and trained observers code-specific parenting behaviours known to predict children's early social, cognitive, and language development. Specifically, the PICCOLO examines four domains of parenting, including behaviours such as affection, responsiveness, encouragement, and teaching. Each domain is scored on a 0–2 Likert scale. The PICCOLO has strong reliability, construct, and predictive validity (37).
• Parent Stress Index—Short Form (PSI-SF): This is a questionnaire completed by parents. It comprises thirty-six items divided into three domains: parental distress, parent-child dysfunctional interaction, and difficult child, which are combined to form a total stress scale (38).

#### 2.5.3 Technological assessment of social behaviour and joint attention

• Video recording of an ESCP through a Kinect Azure camera to collect and analyse quantitative data related to joint attention.

The administration of the ECSP-I will be video-recorded through an Azure Kinect camera to extract gaze orientation and quantitatively assess joint attention automatically. Microsoft Azure Kinect is a Red Green Blue—Depth (RGB-D) time-of-flight camera that allows the 3D identification of 32 body landmarks on the subject's body corresponding to the main anatomical joints, named key points. After the dismission of its predecessor, Kinect V2, the Azure Kinect is considered the most accurate alternative among markerless motion capture devices (39, 40). The sensor will be placed at 1 m and approximately 2 meters from the subjects, defined as the optimal distance for key point acquisition (41). Although children often move during scale administration and their distance from the camera may vary, the proposed setup guarantees video recording of the subjects in most positions of the therapy room. These videos are then processed to extract the gaze direction via a two-step approach inspired by our previous work (42). First, the YOLO (You Only Look Once) algorithm associated with WHENet (Wide Headpose Estimation Network) is applied to segment subjects’ faces in videos (43). The cropped images containing the heads of the subjects are then passed to Gaze360, a machine-learning model that estimates the gaze direction in terms of azimuth and elevation (44, 45). This model can estimate gaze orientation even when faces are completely occluded based on previous and successive frames. Concurrent acquisition of both the subject's position and gaze orientation will ultimately permit the identification of the attention target. In this way, it will be possible to calculate the precise shifting attention times towards the different targets and the total fixation durations, which are valuable measures for quantifying joint attention (46).

• Eye-tracker acquisitions

An experimental Eye-Tracker Screening (ETS) protocol will be implemented using the Tobii Pro Fusion 120 Hz for analysing children's eye movement patterns. Data extraction will be performed using Tobii Pro Lab software. This protocol will measure two components of early social cognition in children: face processing and referential gaze response [i.e., gaze-following mechanism (47, 48)]. This protocol is informed by research indicating that children at high risk for autism exhibit distinct eye movement behaviours concerning the two components mentioned above, particularly when viewing familiar and unfamiliar faces, compared to their typically developing peers (49–51). In this protocol, children watch a set of brief video clips, each lasting approximately five seconds, showing both familiar (e.g., primary caregivers) and unfamiliar individuals [for the identification of unfamiliar faces, see the study by (51)]. The initial set of these videos will comprise static portraits of familiar and unfamiliar faces to assess the children's ability to process faces. The procedure for this evaluation is outlined in detail by Rutherford et al. (52). The evaluation will focus on quantifying the total fixation duration on designated regions of interest, namely, the eyes, nose, and mouth, and mapping the gaze pathways across these facial features. In a randomised order, children will view sets of videos, each approximately 10 s long, to assess their understanding of referential gaze (i.e., gaze following). In these videos, familiar and unfamiliar individuals initially direct their gaze straight ahead for approximately four seconds before shifting their gaze toward one of two objects placed on a table. They will then maintain focus on this object for the following five seconds. The procedure is detailed by Ishikawa & Itakura and Manzi et al. (53, 54). The initial fixation time on the observed object will be analysed to evaluate the gaze-following mechanism, specifically the referential gaze understanding. Furthermore, the protocol aims to quantify the total fixation time on the observed object, thereby providing insights into the level of attention allocated to it by the children.

#### 2.5.4 Biomolecular assessment

Biological samples from family quartets will be collected as follows:

Saliva samples will be collected using 3 different kits for DNA or RNA extraction and protein detection through spongy swabs inserted into the baby's mouth, leading to non-invasive, simple, fast, and, most importantly, painless saliva collection. Saliva samples will be collected from all the probands at T0, T1, and T2, as well as their siblings and parents at T0, to assess parent-to-child genetic transmission and the risk of ASD. If possible, a sample of approximately 40 ml of blood (from the parents) will be collected (see Supplementary Appendix 1).

On these samples, the following analysis will be performed:

• Genetic analysis will be conducted on all enrolled children, their older siblings, and parents using a next-generation sequencing (NGS) gene-targeted panel approach to analyse genes involved in synaptogenesis and immunogenetic regulation.
• Epigenetic analysis of the miRNome in saliva collected from all enrolled children at all time points will be performed by NGS. Epigenetic analysis will allow us to define a panel of microRNAs that can differentiate between ASD children and children with typical development.
• Analysis of inflammatory cytokines and neurotrophic factors using an automated immunoassay system (ELLA, Biotech) will be performed in probands at T0, T1 and T2. The parents will also be characterised for cytokine and lymphocyte subsets at T0.

Correlations between clinical parameters and all biological variables (genetic, epigenetic, inflammatory) will be performed to correlate molecular biomarkers with neurodevelopmental changes at follow-up and before and after intervention.

### 2.6 Sample size

Because of the importance of the ECSP-I in this project as a behavioural outcome tool for obtaining quantitative data through technology, we calculated the sample size to detect associations between the ECSP-I score and the groups (1, 2 and 3) at each time point. The calculation was based on preliminary data (not already published) from the Italian ESCP (ECSP-I) version. A standard deviation of 0.56 is assumed for the ECSP-I scale, and the significance level is *α* = 0.01, obtained by applying the Bonferroni correction for multiple comparisons, performed in pairs between groups, to the type I error; we obtained a sample size of 14 subjects per group to detect a difference of 0.8 between the means of each group using a 2-tailed Student's *t*-test for independent samples, with a power of 80%. Assuming a drop-out rate of 15%, the required sample size increases to 16 subjects per group, that is, 48 subjects in total.

### 2.7 Blinding

Participants and intervention delivery facilitators cannot be blinded to group allocation. However, assessors conducting the outcome measures and coders scoring the video-recorded/audio-recorded observations will be blinded to group allocation.

### 2.8 Interventions

Children allocated in Group 1 (Clinical Monitoring Group) won't perform any treatment, but only clinical evaluations, at the defined time points. On the contrary, children of Groups 2 and 3 will perform training according to the following guidelines.

#### 2.8.1 Active monitoring (AM) group

Children of this group (Group 2) will be enrolled in a 90-minute session once a month for 6 months. Sessions will be performed by an expert neuro and psychomotor therapist for developmental age supported by a child neuropsychiatrist or psychologist. A parent will always be present and actively involved in the intervention. During these sessions, the aims will be (i) to monitor and discuss the achievement of developmental milestones in sensory-motor, cognitive and socio-communication domains through clinical observation; (ii) to share with caregivers some strategies and activities appropriate for the age of the child to promote the achievement of developmental milestones and the active participation of the child in everyday activities; and (iii) to discuss with parents’ needs, problems and concerns about the development of the child and their parent-child relationship to provide educational advice.

#### 2.8.2 Early intervention (EI) group

Children of this group (Group 3) will be enrolled in a 90 min parent-coaching session once a week for six months with an expert neuro and psychomotor therapist for developmental age supported by a child neuropsychiatrist or psychologist. A parent (preferably the mother) will always be present and actively involved in the intervention. The intervention is individualised and tailored to each child's needs, and clinicians will work with parents to identify ways to incorporate objectives into daily life. It will be carried out using a multidimensional, naturalistic and family-centered approach. We will consider promoting postural-motor, socio-communicative and cognitive skills, always considering the sensory profiles of each child. The key elements of the EI protocol are (i) the stimulation of the neurodevelopment of the child in a harmonious way, considering all developmental domains; (ii) the involvement of the caregivers to facilitate social reciprocity and to coach them in understanding the communication signals of their child and to interact with their child in enjoyable, responsive, and non-intrusive modalities; and (iii) the support parents in their parent experience and their feelings about their interaction with their child. During the sessions, the parent's involvement, according to a parent-coaching approach, can guarantee sharing goals and strategies to stimulate the child at home. Parents will also be encouraged to engage in regular stimulation at home, with a goal dose of 20–30 min per day, according to their child's availability. It will be suggested that parents record short home videos each week during play activities to observe and discuss what happened at home. Sessions may also contain a small psychoeducation component on common early parenting challenges such as sleep, crying and feeding.

An explanation of the structures of the AM and EI sessions is reported in Table 1.

**Table 1:** General features and goals of active monitoring (AM) and early intervention (EI) of the ERI-SIBS project.

Component	Active monitoring	Early intervention
Involved professionals	Neuropsychomotor Therapist for Developmental Age + Child Neuropsychiatrist/Child Psychologist	Neuropsychomotor Therapist for Developmental Age + Child Neuropsychiatrist/Child Psychologist
Frequency	1 session of 1,5 h every month	1 session of 1,5 h every week
Role of parents	At least one parent is active in the sessions	At least one parent (preferably the mother) is active in sessions
Goal 1	To provide information about child development and to monitor the achievement of the most important developmental milestones in the child	To stimulate child development in all domains (sensory-motor, cognitive, and social) and to coach the parents in understanding and adequately responding to the communication signals of their child
Goal 2	To share with parents some strategies and activities appropriate for the age of their child for promoting development	To share and implement with parents progressively new strategies and activities for promoting a greater motor and communicative initiative in the child
Goal 3	To discuss with parent's needs, problems, and concerns about the development of the child and their parent-child relationship and to provide educational advice	To support parents in their parent experience and on their feelings about their interaction with their child, to reach together the best strategy for promoting parent-child relationship
Strategies and modalities	Counselling to share and show activities, objects, and strategies to propose to the child during the interaction; discussion and active listening with parents.	Parent-coaching; neuro-psychomotor stimulation of the child; discussion and video-feedback; psychological counselling and active listening for parents.
Instruction for parents at home	Implement strategies and activities during the everyday activities of the child and family.	Stimulations during free play sessions at home, at least for 20–30 min per day; implement strategies and activities also during the everyday activities of the child and family.

### 2.9 Fidelity

The intervention will be carried out by a neuro- and psychomotor therapist for the developmental age, and it will also be supported by a child neuropsychiatrist or psychologist who is an expert in early intervention. The study clinicians are experienced in working with families of children with early neurodevelopmental disabilities. Clinicians will meet every week to provide peer clinical supervision within the context of a multidisciplinary team. All the clinicians will follow the shared protocol. However, some differences in the specific objectives and therapeutic strategies will be based on every child's individual functioning and every family's needs. All the intervention sessions (Groups 2 and 3) will be videotaped and stored on a device owned by the principal investigator. This permits better supervision from the multidisciplinary team.

### 2.10 Patient and public involvement

Preliminary feedback was collected from consumers (parents of children with different developmental disabilities not recruited for this project) on the protocol, the study forms, and the intervention. Consumer feedback was positive, with some changes to the wording made following the input.

### 2.11 Study procedures

Researchers will contact interested parents to assess eligibility and provide detailed study information. Parents will provide written consent before completing baseline assessments (T0), and based on the scores from the primary outcome measures, children will be assigned to the correct group. Infants with no signs of concern (scores at the Griffiths-3 and CSBS-ITC in the normal range), which are siblings of TD children, will be allocated to Group 1 (CM); infants with no signs of concern (scores at the Griffiths-3 and CSBS-ITC in the normal range) which are siblings of ASD children will be allocated to Group 2 (AM); infants with a quotient score below 85 using Griffiths-3 and at least one score above the “concern” cut-off using the CSBS-ITC will be allocated to Group 3 (EI). Families allocated to Groups 2 and 3 will start the intervention within one month of the baseline assessment. Families allocated to Group 1 will perform only the evaluations at different time points. Assessments will be conducted at baseline (T0), which will be when the parents give their consent and within the eight months of life of the child, after six months from the start of the intervention (T1) and after 12 months from the start of the intervention (T2). During the recruitment phase, the study's importance in developing specific guidelines for children at risk of neurodevelopmental disorders will be explained in order to improve adherence to the methodology. Participants may withdraw from the study for any reason at any time.

### 2.12 Data collection and management

The data will be collected, organised, managed, and stored in Research Electronic Data Capture software (REDCap) according to the Italian security, integrity, and confidentiality criteria. The data will be entered into the REDCap database in a potentially individually identifiable format. Once de-identified, the data will be stored in an identifiable format on a secure electronic database protected by the Fondazione Don Gnocchi secure server and accessible only to research team members. The different skills of the staff of the different units involved in the project will be integrated in the best possible way to achieve the desired results. Based on their expertise, different professionals will be coordinated centrally by the neuropsychiatrists to collect several planned measurements and facilitate the adherence and eliciting of information from study participants in a uniform, reproducible manner. The data relating to the project results will be communicated to protect the participants’ privacy. In the case of scientific publications, long-term repositories for anonymised data will be used, which may have open or restricted access criteria to ensure the sustainability of the collected data.

### 2.13 Statistical analysis

Analysis (using STATA or SPSS 28.00) will follow standard methods for trials using comparisons between the three groups. First, a study of the maturation trajectories of the infants will be carried out, with a description of the pre-post intervention changes in the tests carried out. Second, quantitative evaluations of joint attention data and molecular markers will be performed, detected, and described through the mean and standard deviation in the case of a Gaussian distribution of variables or the median and interquartile range in the case of a non-Gaussian distribution. The normality of distributions will be assessed by applying the Shapiro‒Wilk test. For categorical variables, absolute frequencies and percentages will be reported. Genetic analysis of intrafamilial allelic inheritance will be conducted by AFBAC (Affected Family-Based Controls) (55) and TDT (Transmission Disequilibrium Test) (56). The immunological parameters obtained from the groups of ASD children and TD children will be analysed using a nonparametric Mann‒Whitney test that will allow the identification of specific immunological biomarkers of ASD-associated neuroinflammation. Moreover, clinical parameters will be correlated with immunological biomarkers characteristic of ASD children. Finally, epigenetic analysis will allow us to define a panel of microRNAs that can differentiate between ASD children and children with typical development. Using computer algorithms, the potential targets, pathways, and biological functions deregulated by this group of miRNAs will be analysed. These data will also be analysed concerning the clinical parameters evaluated in the study and the genetic and inflammatory characteristics of the subjects studied.

## 3 Discussion

This study aims to provide evidence on the feasibility and efficacy of an innovative and early intervention program for infants at risk of developing neurodevelopmental disorders. In this way, new findings and insights could set the basis for the future development of guidelines on managing early support and intervention for at-risk families, both in the presence and absence of specific risk signs. In fact, evidence suggests that this early and preemptive approach may substantially impact the quality of life for the families involved, reducing parental stress and improving parents’ sense of competence in their role (15, 17). In addition, from an economic standpoint, early diagnosis and intervention can lead to better developmental outcomes, which can result in a decreased need for therapies (such as psychomotor, speech, and physical therapies) and special needs services later in life, thus resulting in improved cost-effectiveness for the health care system in the long run, as already demonstrated (57). Moreover, the approach of the ERI-SIBS program is designed to include in the Italian national health system the more recent scientific literature concerning early intervention for children with neurodevelopmental disorders (58). These are the implementation of training-based interventions harnessing experience-dependent plasticity, the use of environmental enrichment's paradigm for promoting voluntary, self-initiated actions and learning, and the promotion of parent-child interactions, fostering a sense of self-competence and autonomy, a better generalization of child's learnings and the promotion of a secure bonding. Our investigation could contribute to the debate on the intensity and duration of intervention for the developmental domains of young ASD children. In fact, in a recent meta-analysis, Sandbank et al. (59) reported little robust evidence supporting the provision of intensive interventions for this population. Finally, the contribution of biomolecular, genetic, and epigenetic testing, as well as the use of technologies to collect quantitative data on joint attention and early social responses, confers a particular and innovative role to this project: it could allow the collection of preliminary data for characterising biomarkers to understand better not only the natural developmental trajectories of these children but also to identify eventual changes that can be related to clinical early intervention.
