# Supplementary material for: Early Recognition and Intervention in SIBlingS at High Risk for Neurodevelopment Disorders (ERI-SIBS): a controlled trial of an innovative and ecological intervention for siblings of children with autism spectrum disorder
Source: Front Pediatr. 2025 Jan 6;12:1467783. doi: 10.3389/fped.2024.1467783 (PMC11744003; doi:10.3389/fped.2024.1467783)
Supplement: Supplementary file 1 [file Datasheet1.pdf]

APPENDIX 1

Plans for collection, laboratory evaluation, and storage of biological specimens for genetic or molecular analysis in the current trial and for future use in ancillary studies

BACKGROUND AND CHARACTERISTICS OF THE WORKING GROUP

Laboratory of Molecular Medicine and Biotechnologies (LAMMB) – Fondazione Don Gnocchi.

The research activity of LAMMB is in the field of the so-called translational medicine and is therefore aimed at developing new diagnostic and, possibly, therapeutic approaches in the field of neurological diseases with an inflammatory or neurodegenerative aetiology in childhood and adulthood and in the frailty of the elderly. The research activity, therefore, consists of the development and application of advanced biotechnological methods to help identify predisposing factors, events or triggers involved in the aetiology and determinism of different pathologies.

The LAMMB is furnished with equipment for molecular and cellular biology:

NGS genetic analyser (MiSeq, Illumina); droplet digital PCR system (QX200- Bio-Rad); real-time PCR systems: CFX 96/384 Touch (Bio-Rad), AB One-Step (ThermoFisher); fluorometer (Qubit-ThermoFisher); Thermal cyclers; DNA/RNA automatic extractor (Qubit-Qiagen); ChemiDocImaging system (Bio-Rad); Microscope DMi8 Laser Safety (Leica); Flow Sight Imaging FLow-cytometry Amins (Merck); Flow-cytometry FC500, GALLIOS (BeckmanCoulter); Flow-cytometry DXFLEX (BeckmanCoulter); ELISA reader (Sunrise- Tecan); centrifuges; electrophoresis and Western Blot equipment (Bio-Rad); Sonicator (Qnova-WVR); Air flow and Chemical cabinets; CO2 Incubators; Elix MilliQ water purification system.

The LAMMB will be responsible for the genetic epigenetic and immunological analysis required for the scope of the project. As part of the project, the LAMMB will work in collaboration, synergy and continuous dialogue with the other units and, in particular, with the Department of the Child and Adolescence Neuropsychiatry of the IRCCS Don Gnocchi Foundation, from where recruited samples. LAMMB will process all the samples and perform all the necessary experiments according to the most appropriate procedures. The different skills of the staff of the units involved in the project will be integrated in the best possible way in order to achieve the desired results.

PLANS OF BIOLOGICAL SAMPLE COLLECTION

Objective:

Collect saliva samples from probands and their siblings at different time points (T0 and T1) and saliva or blood samples from their parents at T0.

Methods:

Saliva samples are collected using 3 different kits, depending on the final molecular applications: "ORAcollect for paediatrics" (DNAGENOTEK, Ottawa, Canada) for DNA extraction, "ORAcollect RNA" (DNAGENOTEK, Ottawa, Canada) for RNA extraction, and the Saliva's Infant swab (SIS) SalivaBio, (Salimetrics, State College, PA, USA) for cytokine and neurotrophic factor analysis. With these kits,

which use spongy swabs that are inserted into the baby's mouth, saliva collection is non-invasive, simple, fast, and, most importantly, painless. Saliva samples will be collected from the children in the study (at T0, T1) for epigenetic and immunological analysis. Saliva samples will also be collected at the same time, not only from the children in the study but also from their siblings and parents, in order to assess and compare parent-to-child genetic transmission in relation to the risk of ASD. If possible, a sample of approximately 40 mL of blood (from the parents) will be collected for peripheral blood mononuclear cell (PBMC) isolation. The DNA and RNA obtained from the saliva samples will be stored respectively at -20 °C and -80°C and the saliva at -80° C until tested.

PLANS OF IMMUNOLOGIC, GENETIC AND EPIGENETIC ANALYSES

Objective:

Define the genetic, epigenetic and immunological patterns in siblings at high risk for neurodevelopmental disorders, with a focus on the first year of life.

Description of work:

1. Genetic analysis will be conducted by a Next-Generation Sequencing (NGS) gene-targeted panel approach to analyse genes involved in synaptogenesis and immunogenetic regulation for all enrolled children and their parents. Gene association will be reported as genotypes and alleles NUMBER and percentages.
2. Epigenetic analysis of the miRNome of all enrolled children at all time points will be performed by Next-Generation Sequencing. Epigenetic analysis will allow the definition of a panel of microRNAs that can differentiate between children with ASD and children with typical development. MiRNA concentrations will be reported as COPIES/NANOGRAMS of extracted RNA
3. Probands and their ASD siblings will be analyzed for inflammatory cytokines and neurotrophic factors using an automated immunoassay system (ELLA, Biotech, USA) at T0 and T1. Cytokine and neurotrophic factors concentration measured in saliva and plasma samples will be reported as PICOGRAMS/ML. Results calculations will be based on standard curves and internal controls provided by commercial kits. Parents will also be characterised for cytokine profile only at T0.
