# Supplementary material for: Early Recognition and Intervention in SIBlingS at High Risk for Neurodevelopment Disorders (ERI-SIBS): a controlled trial of an innovative and ecological intervention for siblings of children with autism spectrum disorder
Source: Front Pediatr. 2025 Jan 6;12:1467783. doi: 10.3389/fped.2024.1467783 (PMC11744003; doi:10.3389/fped.2024.1467783)
Supplement: Supplementary file 2 [file Datasheet2.pdf]

FOGLIO INFORMATIVO E MODULO DI CONSENSO INFORMATO

Titolo dello studio: **“Riconoscimento e intervento precoce in fratellini/sorelline ad alto rischio per Disturbi del Neurosviluppo (ERI-SIBS)”.**

Al Genitore che esercita la responsabilità genitoriale / Rappresentante legalmente riconosciuto di:

____

(cognome e nome del minore)

Egr. Sig. / Gent.le Sig.ra
La informiamo che stiamo conducendo presso la Fondazione Don Carlo Gnocchi (Onlus), I.R.C.C.S. Santa Maria Nascente (Milano), U.O. Neuropsichiatria e Riabilitazione dell’Età Evolutiva, uno studio dal titolo: *“Riconoscimento e intervento precoce in fratellini/sorelline ad alto rischio per Disturbi del Neurosviluppo (ERI-SIBS)”.*

Per realizzare questo studio desideriamo avvalerci della collaborazione e della disponibilità di suo/a figlio/a /della persona da Lei rappresentata in quanto soddisfa i requisiti scientifici idonei alla partecipazione allo studio. Per questo motivo Le proponiamo di autorizzare la partecipazione di Suo/a Figlio/a della persona da Lei rappresentata al presente studio che sarà condotto sotto la responsabilità della dr.ssa Silvia Annunziata.

Prima che Lei decida se accettare o rifiutare, La invitiamo a leggere con attenzione questo documento, qualora Lei desideri avere ulteriori informazioni e chiarimenti potrà rivolgersi alle D.sse Silvia Annunziata e Anna Cavallini (i cui recapiti sono indicati in fondo al presente documento) responsabile dello studio, che Le dedicherà tutto il tempo necessario per chiarire ogni Suo dubbio, fermo restando che Lei potrà rivolgersi in qualsiasi momento agli operatori coinvolti.
Se Lei ritiene può discutere la proposta contenuta in questo documento – oltre che con noi - anche con il Suo medico di medicina generale, con i Suoi familiari e con altre persone di Sua fiducia. Quindi, può prendersi tutto il tempo necessario per decidere.

Premesse e scopo dello studio
I Disturbi dello Spettro Autistico (ASD) sono Disturbi del Neurosviluppo caratterizzati da anomalie nell’interazione sociale e nella comunicazione verbale e non verbale, e la presenza di interessi ristretti, condotte ripetitive e ipo o iperreattività a stimoli sensoriali a insorgenza precoce.
L’attuale prevalenza di tali disturbi è stimata in crescita. La causa di tali disturbi non è nota, ma sono considerati condizioni con basi neurobiologiche, e con una forte componente genetica. È dimostrato infatti che nelle famiglie in cui è presente un bambino con una diagnosi di Disturbo dello Spettro autistico è più alto il rischio di ricorrenza all’interno della stessa famiglia rispetto alla popolazione generale. Inoltre, studi recenti hanno dimostrato come i fratelli di bambini con diagnosi di ASD incorrano in una percentuale significativamente maggiore di diagnosi di Disturbi del Neurosviluppo rispetto a bambini di famiglie a “basso rischio”, che hanno cioè figli con sviluppo psicomotorio e socio-comunicativo nella norma.
L'attenzione per il riconoscimento precoce è relativa all’importanza che è riconosciuta all’intervento precoce. Un intervento condotto nei primi due anni di vita sembra essere in grado, infatti, di poter modificare la traiettoria dello sviluppo.
Per questo motivo presso l’U.O. di Neuropsichiatria e Riabilitazione dell’età evolutiva di questo centro è stato avviato un protocollo di valutazione e intervento precoce, con inizio nel secondo semestre di vita, in due gruppi di bambini: uno in cui il rischio per Disturbi del Neurosviluppo sia aumentato per la presenza di un fratello/sorella con una diagnosi di Disturbo dello Spettro Autistico, un altro gruppo in cui invece il rischio sia stimato “basso” per la presenza di un fratello/sorella con uno sviluppo in linea con l’età.
Recentemente è stato ipotizzato l’esistenza del cosiddetto “endofenotipo autistico”, ossia di una complessa ragnatela di sintomi comportamentali che correlano con parametri biologici e genetici; uno dei più interessanti fenomeni svelati recentemente in questo ambito di ricerca è che tale endofenotipo è osservabile anche nei fratelli sani, non-autistici, di bambini affetti dalla patologia. In particolare è stato osservato che l’endofenotipo possa estendere i suoi effetti anche su altri parametri, più in dettaglio, sul sistema immunitario che è noto giocare un ruolo preponderante nella neuro infiammazione associata a ASD.
Infine è attualmente noto che particolari condizioni dell’ambiente esterno (inquinamento, stress e infiammazioni materne) possono influenzare il neurosviluppo modulando l’espressione dei geni tramite modificazioni epigenetiche. Tra queste i microRNA rappresentano un promettente ambito di studio per queste patologie, poiché

sono elementi facilmente misurabili in tutti i fluidi biologici, e poiché possono indicare nuove terapie farmacologiche.

Gli obiettivi di questo studio sono quelli di identificare segni di rischio, e di fornire un percorso di supporto al neurosviluppo e intervento precoce per i bambini e le famiglie in cui si riscontrano tali difficoltà; l'obiettivo è inoltre quello di studiare misure quantitative relative alle iniziative socio-comunicative.

Procedure previste dallo studio

- Il presente studio prevede una iniziale raccolta dei dati personali di base (età, sesso) relativi a Suo/a Figlio/a o il minore da Lei rappresentato.
- Sarà effettuata per tutti i partecipanti una prima valutazione al reclutamento, caratterizzata da un minimo di 2 a un massimo di 3 sedute di attività di gioco con il bambino, di cui una sarà videoregistrata, una videoregistrazione di una breve interazione di gioco genitore/tutore-bambino (10 minuti circa), e nella compilazione di alcuni questionari da parte del genitore/tutore.
- Sulla base degli esiti delle valutazioni iniziali verranno costituiti tre gruppi:
    - Nel caso in cui si rilevino difficoltà nello sviluppo psicomotorio o nello sviluppo delle competenze socio-comunicative verrà proposto un percorso di parent coaching. Questo è caratterizzato da una seduta alla settimana, per un totale di 24 sedute (6 mesi circa). Le sedute hanno una durata di 90 minuti. All'interno della seduta verranno proposte alcune attività di gioco, momenti di interazione e di riflessione rispetto a tematiche inerenti la promozione dello sviluppo.
    - Nel caso in cui non si rilevino tali difficoltà e Suo/a Figlio/a /minore da Lei rappresentato ha un fratello/sorella con Disturbo dello Spettro Autistico vengono proposte sedute mensili (6) della durata di 90', per aggiornamento clinico e condivisione di strategie per arricchire l'ambiente e promuovere lo sviluppo.
    - Nel caso in cui non si rilevino tali difficoltà e Suo/a Figlio/a /minore da Lei rappresentato ha un fratello/sorella con sviluppo nella norma non verrà effettuato alcuna altra procedura, se non le rivalutazioni cliniche a 6 e 12 mesi dall'arruolamento.
- Durante la fase valutativa, una camera Kinect posta davanti al bambino, registrerà i movimenti in modo da poterne poi ricavare misure dell'attenzione congiunta.
- Nel corso della partecipazione allo studio ci saranno due momenti di rivalutazione, dopo 6 mesi dall'arruolamento e dopo 12 mesi dall'arruolamento. Tali rivalutazioni consisteranno in due-tre sessioni di gioco con una psicologa/NPI/TNPEE e nella compilazione di alcuni questionari da parte del genitore/tutore.
- All'interno delle sessioni di valutazione verranno mostrati ai bambini su uno schermo brevi video di volti, alternando tra volti di persone a loro familiari e non. I movimenti oculari dei bambini saranno analizzati tramite l'eye-tracker (Tobii Pro Fusion).
- Ai bambini in studio e ai loro fratelli/sorelle verrà prelevato, su base volontaria e non vincolante all'inclusione nel protocollo clinico, un campione di saliva mediante tamponcino boccale. La saliva sarà raccolta sia al tempo di reclutamento che nei due momenti di rivalutazione, utilizzando 3 differenti kit, a seconda delle applicazioni molecolari finali: "ORAcollect for pediatrics" (DNAGENOTEK, Ottawa, Canada) per l'estrazione di DNA, "ORAcollect RNA" (DNAGENOTEK, Ottawa, Canada) per l'estrazione di RNA e il "Saliva's Infant swab (SIS) SalivaBio, (Salimetrics, State College , PA, USA), per le analisi di citochine e dei fattori neurotrofici. Con questi kit che utilizzano tamponcini spugnosi da passare nella bocca del bambino, la raccolta della saliva avviene in modo non invasivo, facile, veloce e soprattutto indolore.
- Ai soli genitori verrà chiesta la disponibilità a fornire un campione di sangue (circa 40 ml) al tempo T0. Se non disponibili al prelievo ematico, verrà chiesta la disponibilità a fornire un campione di saliva.
- Tutti i campioni prelevati saranno consegnati al Laboratorio di Medicina Molecolare e Biotecnologie (LAMMB) dell'IRCCS, dove saranno di prassi contrassegnati con codice anonimo (alfanumerico), e poi processati per l'estrazione di DNA e RNA e cellule. I campioni estratti saranno analizzate presso il Laboratorio per le indagini di tipo biologico previste dallo studio. I campioni verranno conservati alla temperatura di -80°C e-140°C presso il suddetto Laboratorio sotto la responsabilità del Direttore (Prof. Mario Clerici).
- I campioni biologici saranno utilizzati per lo scopo del presente studio senza alcun fine di lucro, essi potranno essere utilizzati per eventuali altre ricerche a lungo termine sempre riguardanti lo studio dei disturbi dello spettro autistico e i cui scopi siano collegati a quelli del presente studio. A tal fine i campioni biologici verranno conservati per 30 anni dopo il termine del progetto presso la bio repository (collezione di campioni di materiale biologico) del LAMMB sotto la responsabilità del prof. Mario Clerici responsabile del Laboratorio suddetto. Qualora vi sarà la possibilità di utilizzare i campioni nell'ambito di progetti i cui scopi e le cui metodologie di ricerca non risultino conformi a quelli descritti nel presente documento, Le sarà chiesto di rilasciare per iscritto un ulteriore specifico consenso. Lei ha il diritto, se lo desidera, di conoscere i risultati delle analisi che la riguardano, quando sia possibile identificare il campione. Questi dati vengono raccolti a scopo di ricerca utilizzando metodi sperimentali. I risultati ottenuti per i pazienti non hanno quindi

valore diagnostico e pertanto non sarà disponibile un referto per le singole famiglie. Il laboratorio ha già raccolto e sta ancora raccogliendo i campioni biologici di famiglie composte da padre, madre, figlio affetto ed eventualmente fratelli, sui quali svolgere gli stessi test genetici. Questi studi denominati di "linkage" riguardano soggetti tra loro geneticamente correlati e sono caratterizzati dalla necessità di avere numerose famiglie in quanto i singoli dati vengono analizzati in modo aggregato. Solo lo studio di tutti i risultati ottenuti da tutte le famiglie potrà fornirci informazioni utili sull'eventuale associazione tra particolari geni e i Disturbi dello Spettro Autistico. Nel caso in cui decida di ritirare dallo studio la persona da Lei rappresentata, campione acquisito sarà immediatamente distrutto e non sarà effettuata nessuna ulteriore valutazione sul campione stesso, salvo che, in origine o a seguito del trattamento, dal campione non sia più possibile risalire alla sua identità.

Possibili benefici

La partecipazione di Suo/a Figlio/a /minore da Lei rappresentato, potrebbe comportare benefici in termini di identificazione precoce di eventuali anomalie o atipie nello sviluppo psicomotorio e socio-comunicativo e di un intervento che possa agire tempestivamente modificando la traiettoria di sviluppo.

La partecipazione di Suo/a Figlio/a /minore da Lei rappresentato potrebbe inoltre comportare benefici in termini di verifica di fattibilità di un protocollo di intervento precoce di parent-coaching che potrà quindi essere integrata nei percorsi riabilitativi e diffusa su più larga scala. La rilevazione dei dati molecolari non comporterà un vantaggio diretto per suo figlio, ma contribuirà ad aumentare le conoscenze sulla malattia dello spettro di autismo ed in particolare identificare biomarcatori di malattia e di efficacia di trattamento riabilitativo.

La partecipazione allo studio NON determina l'esclusione, la riduzione o la sospensione del trattamento ordinario di assistenza e di cura di Suo/a Figlio/a / minore da Lei rappresentato.

Eventuali rischi / effetti collaterali

Le attività da eseguire nel corso dello studio non prevedono l'esecuzione di esami invasivi o di procedure di carattere interventistico, pertanto, la partecipazione per Suo/a Figlio/a/minore da Lei rappresentato non presenta particolari rischi aggiuntivi rispetto a quelli che potrebbero essere correlati all'esecuzione del programma di cura ordinario.

I test iniziali e la successiva applicazione del protocollo avverranno in presenza e sotto la guida e il controllo del personale qualificato (medico, psicologo, terapista della neuropsicomotricità) appartenente alla Fondazione Don Gnocchi.

Per quanto riguarda la raccolta dei campioni biologici, il tamponcino boccale potrebbe causare un leggero fastidio locale risolvibile in pochi minuti. Al termine del prelievo ematico, previsto per i genitori che scelgono questa opzione, che sarà eseguito secondo la comune procedura in uso presso tutti i laboratori di analisi, potrebbe manifestarsi un piccolo ematoma o un livido nella zona del campionamento; per evitare tale inconveniente è consigliabile, alla fine del prelievo, esercitare una lieve pressione sul punto dove è avvenuta la puntura.

Assicurazione

I possibili rischi legati allo svolgimento dello studio, che consiste in attività di ricerca e di sperimentazione, rientrano nella copertura assicurativa prevista dalla polizza generale di assicurazione per responsabilità civile stipulata dalla Fondazione Don Carlo Gnocchi.

Partecipazione allo studio

La partecipazione è completamente libera e volontaria.
Se acconsente a far partecipare Suo/a figlio/a / il minore da Lei rappresentato Le sarà chiesto di firmare, in sua vece, il Modulo di Consenso Informato, allegato al presente documento, prima che la paziente inizi a eseguire la procedura prevista dallo studio.
La firma del modulo allegato è al fine di garantire che Lei abbia ricevuto un'informazione completa e che abbia espresso liberamente la Sua volontà riguardo la partecipazione del minore; tale firma non implica alcun impegno a proseguire lo studio, non costituisce un vincolo di natura contrattuale, né rappresenta una rinuncia ai diritti che spettano alla persona partecipante.
E' possibile, nel caso in cui Lei decida di ritirare il minore dallo studio dopo avere inizialmente accettato, interrompere la partecipazione in qualsiasi momento dandone comunicazione al responsabile dello studio senza dover fornire una giustificazione. La scelta di non far partecipare suo/a figlio/a / il minore da Lei rappresentato o di ritirarla dopo l'iniziale accettazione, non comporta l'esclusione o la limitazione delle cure e dell'assistenza che ella riceve presso le nostre strutture, né alcuna penalizzazione nel rapporto con il personale che lo assiste.

Qualora si venisse a conoscenza di nuovi dati o di risultati che possano influenzare la partecipazione allo studio di Suo/a Figlio/a del minore da Lei rappresentato ne sarà tempestivamente informato/a; inoltre, il responsabile dello studio potrà stabilire il ritiro del minore dallo studio qualora ritenga che tale decisione risponda al suo migliore interesse.
La partecipazione allo studio non determina alcun tipo di onere o di spesa aggiuntiva a Suo carico, inoltre, non è prevista alcuna modifica o sospensione del programma ordinario di assistenza e di cura che Suo/a figlio/a il minore da Lei rappresentato riceve presso la Fondazione.

Per il consenso alla partecipazione del minore allo studio Lei non riceverà alcun compenso economico.

Al termine dello studio, i dati e le informazioni raccolti saranno analizzati per trarne le conclusioni, il promotore e gli sperimentatori si impegnano a renderli disponibili alla comunità scientifica. Lei – se interessato - potrà chiedere al responsabile dello studio presso questo Centro, di comunicarLe i risultati generali della sperimentazione.

Comitato etico

Il Protocollo dello Studio a cui Le viene proposto di partecipare, è stato approvato – unitamente al presente documento - dal Comitato Etico Territoriale Lombardia 4 (C.E. "B-INT" – Fondazione IRCCS Istituto Neurologico Carlo Besta, Milano e Fondazione IRCCS Istituto Nazionale dei Tumori, Milano).
Il Comitato Etico ha, tra le altre cose, verificato che lo studio sia conforme alle Norme di Buona Pratica Clinica e ai principi etici espressi nella Dichiarazione di Helsinki e che la sicurezza, i diritti e il benessere dei partecipanti siano protetti.

Referenti dello Studio

Dr.ssa Silvia Annunziata
Dr.ssa Anna Cavallini
Fondazione Don Carlo Gnocchi (Onlus), I.R.C.C.S. Santa Maria Nascente (Milano),
U.O. Neuropsichiatria e Riabilitazione dell'Età Evolutiva.
Telefono: 02-40308395
E-mail: ancavallini@dongnocchi.it ; sannunziata@dongnocchi.it.

MODULO DI CONSENSO INFORMATO

Titolo dello studio:

"Riconoscimento e intervento precoce in fratellini/sorelline ad alto rischio per Disturbi del Neurosviluppo (ERI-SIBS)"

Io sottoscritto/a: ____
Cognome e Nome in stampatello del padre / madre / rappresentante legalmente riconosciuto.

in qualità di: ____
Indicare il rapporto con il minore (padre, madre, rappresentante legalmente riconosciuto).

di: ____
Cognome e Nome in stampatello del soggetto minore

nato/a a, il: ____
Luogo e data di nascita del soggetto minore

DICHIARO QUANTO SEGUE:

1. ho letto e compreso il foglio informativo per il minore di cui questo modulo è parte integrante;
2. ho avuto la possibilità di porre domande e di chiedere spiegazioni al/alla Dr./D.ssa ____________________________ dal/dalla quale ho ricevuto risposte soddisfacenti;
3. mi sono state illustrate la natura, lo scopo e la durata dello studio, le procedure che saranno seguite, il trattamento previsto per i partecipanti e il tipo di collaborazione che ad essi sarà richiesta;
4. ho compreso che la partecipazione del minore di cui sono il genitore / rappresentante legalmente riconosciuto, è libera e volontaria e che in qualsiasi momento posso decidere di interrompere la sua partecipazione e ritirarlo/a dalla ricerca senza dovere fornire giustificazione e senza che egli/ella sia in alcun modo privato/a di tutta l'assistenza e delle cure di cui ha bisogno e senza che siano compromessi i suoi diritti e il suo rapporto con il medico, con gli operatori sanitari e i tecnici;
5. di volere ☐ di non volere ☐ [barrare l'opzione che interessa]
   che il Medico di base sia informato sulla partecipazione del minore a questo studio.

Tutto ciò premesso, confermo che sono d'accordo alla partecipazione del minore, di cui sono il genitore / rappresentante legalmente riconosciuto, allo studio descritto nel presente documento.

Firma del Genitore / Rappresentante legalmente riconosciuto: ____________________________
[verificata la sua identità]:

Luogo e data: ________________________

Nome e cognome dell'altro genitore (se applicabile): ____
(Cognome e Nome in stampatello)

Dichiaro quanto riportato nei punti 1, 2, 3, 4 del presente modulo tutto ciò premesso, confermo che sono d'accordo alla partecipazione del soggetto minore allo studio.

Firma dell'altro genitore : ________________________ Luogo e data: ________________________
[verificata la sua identità]

PARTE RISERVATA ALL'OPERATORE CHE HA RACCOLTO IL CONSENSO:

Io sottoscritto/a Dr/D.ssa ____ *(Cognome e Nome in stampatello).*

dichiaro:

a. di avere spiegato ai/al genitori/e o al rappresentante legalmente riconosciuto, la natura e lo scopo della ricerca nonché le procedure che saranno adottate, il tipo di collaborazione che sarà richiesta, i possibili rischi, i benefici e le possibili alternative;
b. di avere di avere valutato che le persone sopraindicate, all'atto della sottoscrizione del presente modulo, hanno sufficientemente compreso le informazioni riportate nel presente documento;
c. di non avere esercitato alcuna coercizione o influenza indebita nella richiesta di sottoscrizione del presente modulo di consenso;
d. di rilasciare ai/al genitori/e o al rappresentante legalmente riconosciuto, una copia firmata e datata del presente modulo insieme al foglio informativo.

Luogo e data: ______________________________ Firma: _______________________________

MODULO DI CONSENSO INFORMATO PER:

PRELIEVO, CONSERVAZIONE E UTILIZZO DEL MATERIALE BIOLOGICO PER FINALITÀ DI RICERCA

PER I SOGGETTI MINORI

Io sottoscritto/a: ____

Cognome e Nome in stampatello del padre / madre / rappresentante legalmente riconosciuto.

in qualità di: ____

Indicare il rapporto con il minore (padre, madre, rappresentante legalmente riconosciuto).

di: ____

Cognome e Nome in stampatello del soggetto minore.

nato/a a, il: ____

Luogo e data di nascita del soggetto minore.

dopo essere stato/a informato/a che:

1. presso la Fondazione Don Gnocchi, IRCCS Santa Maria Nascente (Milano) è in corso uno studio dal titolo: ***“Riconoscimento e intervento precoce in fratellini/sorelline ad alto rischio per Disturbi del Neurosviluppo (ERI-SIBS)”*** che richiede per la realizzazione, il prelievo di un campione di saliva;
2. il materiale biologico prelevato verrà utilizzato dal personale del IRCCS Santa Maria Nascente (Milano) esclusivamente per le finalità di ricerca legate all’esecuzione dello studio: ***“Riconoscimento e intervento precoce in fratellini/sorelline ad alto rischio per Disturbi del Neurosviluppo (ERI-SIBS)”,*** senza alcun fine di lucro e previa approvazione del suddetto studio da parte del Comitato Etico Territoriale competente;
3. il materiale biologico prelevato verrà conservato in condizioni di sicurezza secondo procedure scientificamente riconosciute e al termine dello studio verrà distrutto;
4. qualora vi fosse l’eventuale possibilità di utilizzare - sia presso Fondazione Don Gnocchi, IRCCS Santa Maria Nascente (Milano) sia, eccezionalmente, presso altre strutture - il materiale biologico prelevato nell’ambito di progetti i cui scopi e le cui metodologie di ricerca non risultino conformi a quelli descritti nel foglio informativo del presente studio, mi sarà chiesto di rilasciare per iscritto un ulteriore specifico consenso;
5. il materiale biologico verrà reso anonimo in modo che non sia possibile, se non al responsabile dello studio, risalire alle generalità della persona da me rappresentata;
6. avrò il diritto, nel caso in cui lo richieda, di conoscere i risultati dello studio;
7. verrà garantita la tutela della riservatezza dei dati personali in conformità con le disposizioni vigenti e con quanto precedentemente indicato nel relativo paragrafo del foglio informativo;
8. il presente consenso potrà essere in ogni momento revocato, in tal caso il campione di materiale biologico prelevato, se ancora conservato, sarà immediatamente distrutto, salvo che - in origine o a seguito del trattamento - dal campione non sia più possibile risalire all’identità del soggetto minore;

DICHIARO:

- di essere stato/a chiaramente ed esaurientemente informato/a sull’attività di studio proposta e sulle esigenze che rendono indispensabile il prelievo, l’utilizzo e la conservazione del materiale biologico per le finalità dello studio e per il tempo strettamente necessario alla sua esecuzione;

- di prestare il consenso ☐ di non prestare il consenso ☐ *[barrare l'opzione che interessa]*
  al prelievo del materiale biologico del materiale biologico appartenente al soggetto minore e al suo trattamento esclusivamente per le finalità di ricerca scientifica legate allo studio: ***"Riconoscimento e intervento precoce in fratellini/sorelline ad alto rischio per Disturbi del Neurosviluppo (ERI-SIBS)"*** e per il tempo necessario alla sua esecuzione;

- di autorizzare ☐ di non autorizzare ☐ *[barrare l'opzione che interessa]*
  il Centro IRCCCS Santa Maria Nascente (Milano) a conservare il campione biologico appartenente al soggetto minore presso la biorepository del LAMMB esclusivamente per le finalità di ricerca scientifica legate allo studio: ***"Riconoscimento e intervento precoce in fratellini/sorelline ad alto rischio per Disturbi del Neurosviluppo (ERI-SIBS)",*** e per il tempo necessario alla sua esecuzione;

- di prestare il consenso ☐ di non prestare il consenso ☐ *[barrare l'opzione che interessa]*
  alla conservazione e all'ulteriore trattamento del campione biologico appartenente al soggetto minore per lo svolgimento di studi diversi a condizione che l'utilizzo avvenga nell'ambito di progetti i cui scopi e le cui metodologie di ricerca risultino conformi a quelli descritti nel foglio informativo del presente studio e che siano stati approvati dal Comitato Etico Territoriale competente;

- di volere ☐ di non volere ☐ *[barrare l'opzione che interessa]*
  essere informato/a dei risultati sullo studio dei campioni biologici riferiti alla persona da me rappresentata, compatibilmente con i tempi necessari per la conclusione dello studio;

- di essere disponibile a sottoporsi al prelievo di un campione biologico di:
  sangue ☐ *oppure, in alternativa, di:* saliva ☐ *[barrare l'opzione che interessa]*

Firma del Genitore / Rappresentante legalmente riconosciuto: ____
[verificata la sua identità]:

Luogo e data: ____________________________

Cognome e Nome dell'altro genitore (se applicabile): ____
Cognome e Nome in stampatello

Dichiaro quanto riportato nei punti 1, 2, 3, 4, 5, 6, 7 e 8 del presente modulo tutto ciò premesso, confermo che sono d'accordo alla partecipazione del soggetto minore al progetto di ricerca.

Firma dell'altro genitore: ___________________________ Luogo e data: _______________________
[verificata la sua identità]

PARTE RISERVATA ALL'OPERATORE CHE HA PRESENTATO L'INFORMATIVA

Io sottoscritto/a Dr/D.ssa ____ *(Cognome e Nome in stampatello)*

DICHIARO:

a. di avere informato i/il genitori/e o il rappresentante legalmente riconosciuto in merito a quanto descritto ai precedenti punti 1, 2, 3, 4, 5, 6, 7 e 8;

b. di avere di avere valutato che le persone sopraindicate, all'atto della sottoscrizione del presente modulo, hanno sufficientemente compreso le informazioni riportate nel presente documento;

c. di non avere esercitato alcuna coercizione o influenza indebita nella richiesta di sottoscrizione del presente modulo di consenso;

d. di rilasciare ai/al genitori/e o al rappresentante legalmente riconosciuto, una copia firmata e datata del presente modulo.

Luogo e data: ____________________________ Firma: _____________________________

Nr. Codice assegnato al soggetto minore partecipante: ____________________________
